# Long-Term Exposure to Traffic-Related Air Pollution Associated with Blood Pressure and Self-Reported Hypertension in a Danish Cohort

**DOI:** 10.1289/ehp.1103631

**Published:** 2012-01-03

**Authors:** Mette Sørensen, Barbara Hoffmann, Martin Hvidberg, Matthias Ketzel, Steen Solvang Jensen, Zorana Jovanovic Andersen, Anne Tjønneland, Kim Overvad, Ole Raaschou-Nielsen

**Affiliations:** 1 Institute of Cancer Epidemiology, Danish Cancer Society, Copenhagen, Denmark; 2 IUF-Leibniz Research Institute for Environmental Medicine and Medical Faculty, Heinrich Heine University of Düsseldorf, Düsseldorf, Germany; 3 National Environmental Research Institute, Aarhus University, Roskilde, Denmark; 4 Department of Epidemiology, School of Public Health, Aarhus University, Aarhus, Denmark; 5 Department of Cardiology, Centre for Cardiovascular Research, Aalborg Hospital, Aarhus University Hospital, Aalborg, Denmark

**Keywords:** air pollution, blood pressure, hypertension, epidemiology, nitrogen oxide

## Abstract

Background: Short-term exposure to air pollution has been associated with changes in blood pressure (BP) and emergency department visits for hypertension, but little is known about the effects of long-term exposure to traffic-related air pollution on BP and hypertension.

Objectives: We studied whether long-term exposure to air pollution is associated with BP and hypertension.

Methods: In 1993–1997, 57,053 participants 50–64 years of age were enrolled in a population-based cohort study. Systolic and diastolic BP (SBP and DBP, respectively) were measured at enrollment. Self-reported incident hypertension during a mean follow-up of 5.3 years was assessed by questionnaire. We used a validated dispersion model to estimate residential long-term nitrogen oxides (NO_x_), a marker of traffic-related air pollution, for the 1- and 5-year periods prior to enrollment and before a diagnosis of hypertension. We conducted a cross-sectional analysis of associations between air pollution and BP at enrollment with linear regression, adjusting for traffic noise, measured short-term NO_x_, temperature, relative humidity, and potential lifestyle confounders (*n* = 44,436). We analyzed incident hypertension with Cox regression, adjusting for traffic noise and potential confounders.

Results: A doubling of NO_x_ exposure during 1- and 5-year periods preceding enrollment was associated with 0.53-mmHg decreases [95% confidence interval (CI): –0.88, –0.19 mmHg] and 0.50-mmHg decreases (95% CI: –0.84, –0.16 mmHg) in SBP, respectively. Long-term exposure also was associated with a lower prevalence of baseline self-reported hypertension (per doubling of 5-year mean NO_x_: odds ratio = 0.96; 95% CI: 0.91, 1.00), whereas long-term NO_x_ exposure was not associated with incident self-reported hypertension during follow-up.

Conclusions: Long-term exposure to traffic-related air pollution was associated with a slightly lower prevalence of BP at baseline, but was not associated with incident hypertension.

Exposure to particulate matter (PM) air pollution has been associated with myocardial infarction and stroke (Brook et al. 2010). The mechanisms believed to be involved include alteration of the autonomic function of the heart, vascular reactivity, induction of systemic inflammation, and endothelial dysfunction (Brook et al. 2010), which in turn may affect blood pressure (BP) and the risk of hypertension. It has therefore been hypothesized that high levels of air pollution may increase BP and the risk of hypertension.

Studies investigating associations between air pollution and BP have focused mainly on short-term effects, with some studies reporting small increases in systolic and diastolic BP (SBP and DBP, respectively) (Brook et al. 2009; Chuang et al. 2010; de Paula et al. 2005; Dvonch et al. 2009; Urch et al. 2005; Zanobetti et al. 2004) and others reporting no association (Madsen and Nafstad 2006) or even inverse associations (Brauer et al. 2001; Harrabi et al. 2006; Ibald-Mulli et al. 2004). Two studies have investigated associations between longer-term air pollution exposure and BP (Auchincloss et al. 2008; Chuang et al. 2011). In a cross-sectional study of approximately 5,000 persons 45–84 years of age, Auchincloss et al. (2008) reported that the 30-day mean of PM_2.5_ (PM ≤ 2.5 μm in aerodynamic diameter) was positively associated with SBP, whereas the association with DBP was weaker and statistically insignificant. In a cross-sectional study of 1,023 elderly persons, Chuang et al. (2011) reported that systolic and DBP both were highly correlated with yearly mean levels of several pollutants.

Little is known about the effects of air pollution on hypertension. Short-term exposure to air pollution has been positively associated with emergency department visits for hypertension (Guo et al. 2010a, 2010b), and a recent study reported a significant positive association between estimated annual exposure to residential PM_2.5_ and the prevalence of self-reported hypertension (Johnson and Parker 2009). No studies have investigated the effects of long-term air pollution on the incidence of hypertension.

Residential exposure to air pollution and road traffic noise are positively correlated (de Kluizenaar et al. 2007; Sørensen et al. 2011). Because exposure to traffic noise has been associated with changes in BP and hypertension (Babisch 2006; de Kluizenaar et al. 2007), road traffic noise is a potentially important confounder in air pollution studies.

In this study we tested the hypothesis that long-term exposure to traffic-related air pollution increases systolic and DBP and the prevalence and risk of hypertension, independent of short-term exposure to air pollution and exposure to road traffic noise.

## Methods

*Study population.* The study was based on the Diet, Cancer and Health cohort (Tjønneland et al. 2007). In total, 57,053 of 160,725 residents of Copenhagen or Aarhus 50–64 years of age without a history of cancer (excluding nonmelanoma skin cancer) were enrolled into the original cohort between 1993 and 1997. An invitation to participate in a follow-up survey was mailed with a follow-up questionnaire to the 54,379 living cohort members still residing in Denmark in 2000–2002. The response rate was 83.3%, corresponding to 45,271 participants. All study participants provided written informed consent. The study was conducted in accordance with the Declaration of Helsinki (World Medical Association 2008) and approved by local ethical committees.

At baseline enrollment into the original cohort study, each participant completed a self-administered questionnaire that included questions on lifestyle habits, health status, whether they suffered or had ever suffered from hypertension, and whether they received or had ever received medication for hypertension.

*Exposure assessment.* Using the Danish AirGIS modeling system, we modeled nitrogen oxides (NO_x_), nitrogen dioxide (NO_2_), and nitrogen oxide (NO) concentrations in the air at each address at which the cohort members lived from 5 years prior to baseline until follow-up was completed in 2000–2002. AirGIS calculates air pollution at a location as the sum of local air pollution from street traffic [calculated with the Operational Street Pollution Model from input data on traffic (intensity and type), emission factors, street and building geometry, and meteorology (Berkowicz 2000; Kakosimos et al. 2011)]; urban background [from a simplified area source dispersion model that takes into account urban vehicle emission density, city dimensions (transport distance), and building height (Berkowicz et al. 2008)]; and regional background estimated from trends at rural monitoring stations and national vehicle emissions. Input data have been described elsewhere (Raaschou-Nielsen et al. 2010). The AirGIS system has been validated in several studies, and the correlation (*r*) between modeled and measured half-year mean NO_2_ concentrations at 204 positions in the greater Copenhagen area was 0.90 (Ketzel et al. in press; Raaschou-Nielsen et al. 2000). The AirGIS system calculates air pollution hour by hour, which was summarized as the yearly average concentration at each residential address.

We used NO_x_ as a measure of exposure to air pollution from traffic because measured NO_x_ correlates strongly with other traffic-related pollutants in Danish streets: *r* = 0.93 for total particle number concentration (10–700 nm) and *r* = 0.70 for PM_10_ (PM ≤ 10 μm in aerodynamic diameter) (Hertel et al. 2001; Ketzel et al. 2003). If NO_x_, NO_2_, and NO could not be calculated because of failed geocoding, we imputed the concentration calculated at the preceding or subsequent residential address of the cohort member as previously described (Raaschou-Nielsen et al. 2011). We then calculated 1-year and 5-year time-weighted average NO_x_, NO_2_, and NO concentrations before baseline enrollment (cross-sectional study), and 1-year and 5-year time-weighted averages before a new diagnosis of hypertension or the end of follow-up (follow-up study).

Based on the enrollment address and the geographical information system (GIS) road network, we generated two additional traffic variables: a dichotomous indicator for the presence or absence of a street with a traffic density > 10,000 vehicles per day within 50 or 100 m of the residence, and the total number of kilometers driven by vehicles within 200 m of the residence each day (the product of street length and traffic density for all streets within a 200-m radius).

We used hourly measurements at a urban background monitoring station (20 m above ground; chemiluminiscence NO/NO_x_ model 200A; Teledyne Advanced Pollution Instrumentation, San Diego, CA, USA) to estimate 3-day average exposures to NO_x_, NO_2_, and NO (on the day of the BP measurement and the 2 preceding days) among participants enrolled by the Copenhagen center. The monitoring station was located in the center of Copenhagen, with a median residential distance from the monitoring station of 5.5 km (5th–95th percentile, 1.5–14.2 km). We used hourly measures of temperature and relative humidity from three locations (Copenhagen, Aalborg, and Odense) to estimate 3-day averages for all participants. Previous studies on air pollution and BP have found different lags and cumulative exposures to be important (Auchincloss et al. 2008; Dvonch et al. 2009; Harrabi et al. 2006; Zanobetti et al. 2004). We calculated a 3-day mean because this has been suggested to be related to BP (Dvonch et al. 2009; Zanobetti et al. 2004).

We estimated exposure to road traffic noise (*L*_den_) at all enrollment addresses using the computer software SoundPLAN [http://www.soundplan.dk/; see Supplemental Material (http://dx.doi.org/10.1289/ehp.1103631)].

*BP measurement.* At baseline enrollment, trained staff members measured brachial artery BP tomated TAKEDA UA 751 or UA-743 using automated oscillometric sphygmomanometers (model UA 751 or UA-743; Takeda Pharmaceutical Co. Ltd., Osaka, Japan). The measurement was conducted with the subject in the supine position after a minimum of 5 min rest and at least 30 min after tobacco smoking and intake of food, tea, or coffee. If SBP was ≥ 160 mmHg more, or if DBP was ≥ 95 mmHg, the measurement was repeated after an interval of at least 3 min, and the lower of the two measurements was used. We excluded from the present analysis all participants who indicated on the enrollment questionnaire that they were taking or had ever taken medication for hypertension. Height and weight were measured at baseline according to standardized protocols.

*Incidence of hypertension.* Information on hypertension was assessed by questionnaire at enrollment and in the follow-up survey. Specifically, at enrollment participants were asked whether they had ever been hypertensive or were taking or had ever taken hypertension medication, and in the follow-up survey they were asked whether they had ever been diagnosed with hypertension by a medical doctor or were taking or had ever taken hypertension medication. In both the cross-sectional study on hypertension and the follow-up study, we excluded all participants with hypertension at or prior to enrollment and participants with missing or contradictory answers to the hypertension questions.

*Statistical methods.* Cross-sectional analysis of BP and hypertension. We used general linear models to estimate associations between residential exposure to long-term NO_x_, NO_2_, and NO (1- and 5-year averages prior to baseline) and systolic and DBP measured at baseline (among participants who did not report use of medications to treat hypertension), and logistic regression models to estimate associations between 1- and 5-year average NO_x_, NO_2_, and NO concentrations and the prevalence of self-reported hypertension at baseline (PROC GLM and PROC GENMOD in SAS, version 9.1; SAS Institute Inc., Cary, NC, USA). Exposures were modeled as categorical variables (with cut points based on quartiles) and as continuous variables after logarithmic transformation (log_2_) to satisfy the assumption of linearity, which we evaluated using linear spline models with boundaries at deciles of exposure for the analytic cohort (BP) or cases (hypertension) (Greenland 1995). In addition, we estimated associations of BP and prevalent hypertension with short-term NO_x_, NO_2_, and NO exposures averaged over 3 days (the day of BP measurement and the previous 2 days, log_2_ transformed and categorical) among Copenhagen residents, and associations with the presence or absence of a major road within 50 m of the baseline residence and traffic density within 200 m of the baseline residence (log_2_ transformed or categorical) among all participants.

We adjusted analyses for potential confounders: age (continuous), sex, calendar year, center of enrollment (Copenhagen or Aarhus), area [Copenhagen city, Aarhus city, or Copenhagen or Aarhus surroundings (defined as residence within 7–25 km of either city center)], length of school attendance (< 8, 8–10, > 10 years), body mass index (BMI; kilograms per meter squared, linear), smoking status (never, former, current), alcohol intake (yes/no; grams per day among drinkers, linear), intake of fruit and vegetables (linear splines with a knot at 350 g/day), sport during leisure time (yes/no; hours per week among active, continuous), road traffic noise (*L*_den_; decibels; residential exposure at enrollment), season (winter, spring, summer, and autumn), mean relative humidity (continuous), and ambient temperature during 3 days (the day of BP measurement and the 2 preceding days). Temperature showed a weak inverse association with BP ≤ 11.5°C and a steep inverse association at temperatures > 11.5°C. Therefore, temperature was modeled using linear splines with a knot at 11.5°C. In addition we adjusted for the socioeconomic status (SES) of the participants’ municipality (or district for Copenhagen residents) classified as low, medium, or high based on information on average education, work market affiliation, and income at the time of enrollment. Analysis of associations with short-term NO_x_, NO_2_, and NO concentrations were also adjusted by the 1-year mean concentration of NO_x_, NO_2_, or NO, respectively, in the previous year.

In a secondary analysis restricted to Copenhagen residents (*n* = 21,507), we adjusted associations between long-term NO_x_ by measured ambient NO_x_ concentrations averaged over the day of the BP measurement and the previous 3 days. In addition, we conducted sensitivity analyses restricted to participants with normal BP (SBP ≤ 140 and/or DBP ≤ 90) or participants with SBP < 160 and/or DBP < 100.

In exploratory analyses, we tested for interactions between modeled long-term exposure to NO_x_ and sex, education, smoking, temperature, area, SES, and history of cardiovascular disease by introducing interaction terms into the model.

Graphical presentation of the functional form of association between NO_x_ and SBP adjusted for the potential confounders was estimated with the OLS function in Design Library[R statistical software, version 2.9.0 (http://www.r-project.org/).

Follow-up for hypertension. We analyzed data based on Cox proportional hazards model with age as the underlying time metric (Thiebaut and Benichou 2004). We used left truncation at age of enrollment, so that subjects were considered at risk from enrollment into the cohort, and right censoring at age of event (self-reported hypertension) or age at follow-up survey, whichever came first. We stratified all analyses by sex and calendar year. Exposure to long-term air pollution was modeled using time-dependent variables of time-weighted average NO_x_, NO_2_, and NO concentrations at each year of age during follow-up (one row of data for each year of age that a participant contributed to follow-up).

We calculated incidence rate ratios (IRRs) for hypertension in association with 1- and 5-year mean NO_x_, NO_2_, and NO concentrations at the time of diagnosis compared with 1- and 5-year mean NO_x_, NO_2_, and NO concentrations for all cohort members at risk at that point in time. IRRs for the two traffic proxies (major road and traffic load) were calculated using enrollment addresses. Analyses were adjusted for baseline information on smoking status, length of school attendance, alcohol intake, intake of fruit and vegetables, BMI, sport during leisure time, SES, area, and traffic noise. We interpreted a *p*-value < 0.05 as statistically significant.

## Results

*BP and baseline hypertension.* Of 57,053 participants, we excluded 571 who had been diagnosed with cancer before baseline, but because of delay in the Danish Cancer Registry, were erroneously included; 2,737 with incomplete residential address information; 63 without BP measurement; and 2,961 with missing information on covariates leaving 50,721 participants for the baseline hypertension analyses. Of these, 6,285 received hypertension medicine at and/or before enrollment, leaving 44,436 participants for the BP analyses.

Table 1 shows the distribution of baseline characteristics in the study population. Long-term exposure to NO_x_ and traffic load at the address at enrollment was correlated, with a Spearman rank coefficient (*r*_S_) of 0.95 between the 1- and 5-year mean NO_x_ (*p* < 0.0001) and 0.51 between traffic load and 1-year NO_x_ mean (*p* < 0.0001). Modeled exposure to NO_x_, NO_2_, and NO at the enrollment address was highly correlated: 0.98 between NO_x_ and NO, 0.97 between NO_x_ and NO_2_, and 0.92 between NO_2_ and NO (1-year data; *p* < 0.0001). Short- and long-term exposure to NO_x_ were not correlated. There was a significant correlation between long-term exposure to NO_x_ and *L*_den_ at enrollment (0.69 and 0.67 for the 1- and 5-year period preceding enrollment; *p* < 0.0001).

**Table 1:** Baseline characteristics of the study populations.

Baseline			Follow-up		
Characteristic at enrollment	Percent (*n*)	Median (5th–95th percentile)	Percent (*n*)	Median (5th–95th percentile)
All		100 (44,436)				100 (33,275)		
Age (years)				55.9 (50.7–64.1)				55.8 (50.7–64.0)
Sex								
Women		52 (23,092)				53 (17,526)		
Men		48 (21,344)				47 (15,749)		
Years of education								
≤ 7		33 (14,467)				31 (10,257)		
8–10		46 (20,443)				47 (15,495)		
≥ 11		21 (9,526)				23 (7,523)		
Municipality SES*a*								
Low		14 (6,268)				14 (4,517)		
Medium		64 (28,573)				64 (21,254)		
High		22 (9,595)				23 (7,504)		
Area								
Copenhagen city		26 (11,486)				25 (8,258)		
Aarhus city		29 (13,028)				29 (9,648)		
Copenhagen/Aarhus surroundings		45 (19,922)				46 (15,369)		
BMI (kg/m^2^)				25.3 (20.4–32.8)				25.1 (20.3–32.2)
Smoking								
Never		36 (15,824)				37 (12,395)		
Former		28 (12,258)				28 (9,471)		
Current		37 (16,354)				34 (11,409)		
Drinking alcohol								
No		2 (958)				2 (585)		
Yes (g/day)		98 (43,478)		13.1 (1.00–60.5)		98 (32,690)		13.2 (1.20–61.6)
Intake of fruit and vegetables (g/day)				346 (108–798)				351 (115–799)
Physical activity								
No		45 (19,902)				42 (14,085)		
Yes (hr/week)		55 (24,534)		2.0 (0.5–7.0)		58 (19,190)		2.0 (0.5–6.5)
NO_x_ measure (µg/m^3^)*b*								
NO_x_, 1 year				20.2 (14.3–86.8)				20.0 (14.3–85.4)
NO_x_, 5 year				19.6 (14.3–87.5)				19.3 (14.3–85.7)
NO_2_, 1 year				16.3 (12.0–32.6)				16.2 (12.4–32.5)
NO_2_, 5 year				15.6 (11.9–32.7)				15.6 (12.0–32.2)
NO, 1 year				3.64 (2.19–54.3)				3.62 (2.23–52.3)
NO, 5 year				4.03 (2.24–55.3)				3.79 (2.34–53.9)
Major road*c* within 50 m								
No		92 (40,973)				92 (30,763)		
Yes		8 (3,462)				8 (2,512)		
Traffic load within 200 m (10^3^ vehicle km/day)				2.46 (0.27–15.0)				2.34 (0.27–14.9)
Road traffic noise (*L*_den_, dB)				56.3 (48.4–70.0)				56.1 (48.3–69.9)
Baseline SBP (mmHg)				136 (108–172)				134 (108–166)
Baseline DBP (mmHg)				81 (67–100)				80 (66–96)
**a**Based on municipality information on education, work market affiliation, and income. **b**Time-weighted average concentration at residences at and before enrollment. **c**More than 10,000 vehicles per day.								

The distributions of systolic and DBP were slightly right-skewed. However, similar results were observed for untransformed and log-transformed values, and regression estimates for the untransformed data are presented.

**Figure 1:**
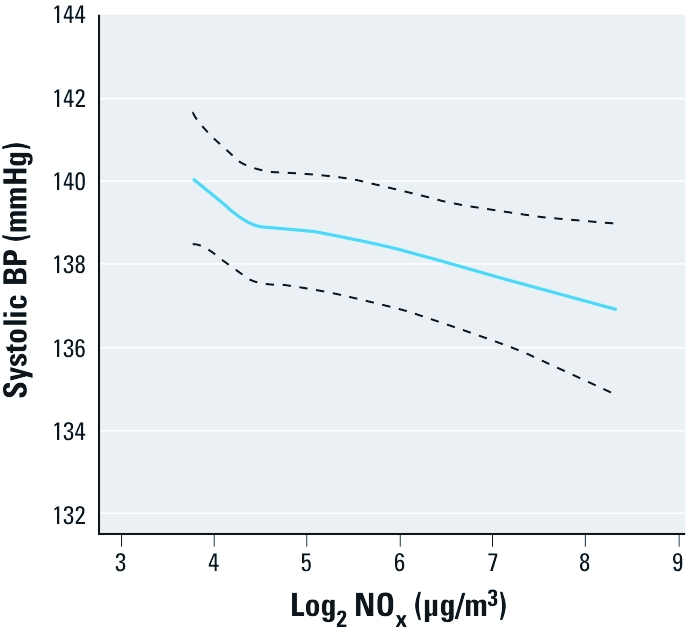
Association [mean (95% CI)] between NO_x_ exposure 5 years preceding enrollment (log transformed) and SBP in 44,436 cohort participants, adjusted for age, sex, center, calendar year, area, smoking, BMI, length of school attendance, municipality SES, intake of alcohol, fruit and vegetables, physical activity, traffic noise, season, temperature, and relative humidity.

Long-term exposure to NO_x_ was inversely associated with BP (Figure 1, Table 2). Although significant, the estimated changes were rather small. Categorical analyses showed a monotonically inverse dose–response relationship between the 1- and 5-year NO_x_ means and SBP, whereas this was not apparent for the DBP. Corresponding estimates for long-term exposures to NO_2_ and NO were generally consistent with those shown for NO_x_ [see Supplemental Material, Table 1 (http://dx.doi.org/10.1289/ehp.1103631)].

**Table 2:** Associations between concentrations of NO_x_ and traffic at the residence and systolic and DBP at enrollment [mean (95% CI)].

Difference in BP (mmHg)*a*		
Exposure	*n*	Systolic	Diastolic
NO_x_ 5-year mean (µg/m^3^)*b*^,c^						
< 16.9		11,109		0.00		0.00
16.9–19.6		11,109		–0.20 (–0.72, 0.32)		–0.05 (–0.32, 0.23)
19.6–28.2		11,109		–0.49 (–1.08, 0.09)		–0.39 (–0.70, –0.08)
> 28.2		11,109		–0.62 (–1.35, 0.11)		–0.32 (–0.71, 0.07)
Linear trend per doubling		44,436		–0.50 (–0.84, –0.16)		–0.24 (–0.42, –0.07)
NO_x_ 1-year mean (µg/m^3^)*b*^,c^						
< 17.0		11,116		0.00		0.00
17.0–20.2		11,102		–0.32 (–0.86, 0.22)		0.01 (–0.28, 0.29)
20.2–27.2		11,109		–0.49 (–1.08, 0.11)		–0.32 (–0.63, –0.00)
> 27.2		11,109		–0.58 (–1.32, 0.15)		–0.24 (–0.62, 0.15)
Linear trend per doubling		44,436		–0.53 (–0.88, –0.19)		–0.24 (–0.42, –0.06)
NO_x_, 3-day mean*b*^,d^						
< 15.2		5,360		0.00		0.00
15.2–19.8		5,419		–1.29 (–2.01, –0.58)		–0.51 (–0.89, –0.13)
19.8–27.6		5,353		–0.81 (–1.54, –0.09)		–0.44 (–0.82, –0.05)
> 27.6		5,375		–1.12 (–1.88, –0.35)		–0.41 (–0.81, 0.00)
Linear trend per doubling		21,507		–0.50 (–0.93, –0.07)		–0.25 (–0.48, –0.02)
Major road*e* within 50 m*f*						
No		40,973		0.00		0.00
Yes		3,462		–0.80 (–1.53, –0.06)		–0.51 (–0.89, –0.12)
Traffic load within 200 m (10^3^ vehicle km/day)*b*^,f^						
< 0.85		11,109		0.00		0.00
0.85–2.46		11,110		0.27 (–0.21, 0.76)		0.05 (–0.21, 0.30)
2.46–6.54		11,109		–0.69 (–1.21, –0.17)		–0.13 (–0.41, 0.14)
> 6.54		11,108		–0.60 (–1.22, 0.01)		–0.21 (–0.53, 0.12)
Linear trend per doubling		44,436		–0.10 (–0.22, 0.02)		–0.03 (–0.09, 0.03)
**a**Adjusted by age, sex, center, calendar year, area, smoking status, BMI, length of school attendance, municipality SES, alcohol intake, intake of fruit and vegetables, physical activity, traffic noise, season, temperature, and relative humidity. **b**The cutoff points between exposure groups were the 25th, 50th, and 75th percentiles. **c**Time-weighted average concentration of NO_x_ 1 and 5 years preceding enrollment. **d**Based on participants from the Copenhagen center. The analysis of 3-days mean of NO_x_ was also adjusted by the preceding 1-year mean concentration of NO_x_. **e**More than 10,000 vehicles per day. **f**At enrollment.						

Adjustment for road traffic noise changed the estimates slightly (data not shown); for example, a doubling in 1-year NO_x_ was associated with a –0.39 mmHg change in SBP before adjustment for traffic noise and a –0.53 mmHg change in SBP after adjustment. Further adjustment by short-term NO_x_ concentrations (among Copenhagen participants only) had little effect on estimates (data not shown). For example, the estimated changes in SBP per doubling of 1- and 5-year NO_x_ exposures were –0.50 mmHg [95% confidence interval (CI): –0.93, –0.07 mmHg] and –0.51 mmHg (95% CI: –0.94, –0.08 mmHg), respectively, after adjusting for short-term NO_x_. When restricted to the 23,982 participants who had normal BP at baseline, the estimated changes in SBP per doubling of 1- and 5-year NO_x_ exposures were –0.24 mmHg (95% CI: –0.50, 0.01 mmHg) and –0.20 mmHg (95% CI: –0.45, 0.05 mmHg), respectively. When restricted to the 38,565 participants who had SBP < 160 and/or DBP < 100, the corresponding estimates were –0.27 mmHg (95% CI: –0.55, 0.01 mmHg) and –0.22 mmHg (95% CI: –0.50, 0.05 mmHg), respectively.

Inverse associations between exposure and BP were also estimated for short-term NO_x_ and for the two traffic proxies (Table 2). The categorical analyses for the 3-day NO_x_ mean and traffic load showed no clear dose–response relationship in relation to BP.

Sex, temperature, and a diagnosis of cardiovascular disease appeared to modify the association between NO_x_ and SBP (Table 3). The inverse relationship between exposure and BP seemed stronger in women than in men and was only apparent at temperatures below 15°C and among participants without cardiovascular disease, whereas among participants with a history of cardiovascular disease there seemed to be a positive association. Furthermore, for measured short-term NO_x_ (3-day mean) we found changes of –0.27 mmHg (95% CI: –1.73, 1.39 mmHg) and –0.56 mmHg (95% CI: –1.00, –0.11 mmHg) for above and below 15°C, respectively, per doubling in 3-day NO_x_ mean (*p* for interaction = 0.71).

**Table 3:** Modification of associations between concentrations of NO_x_ and SBP by baseline characteristics [mean (95% CI)].

Difference in SBP (mmHg)				
Covariate	*n*	Per doubling in 1-year NO_x_	*p*-Value	Per doubling in 5-year NO_x_	*p*-Value
Sex					0.003			0.003
Women		23,092		–0.83 (–1.23, –0.43)			–0.80 (–1.19, –0.41)	
Men		21,344		–0.18 (–0.60, 0.24)			–0.16 (–0.57, 0.24)	
Years of education					0.18			0.11
≤ 7		14,467		–0.34 (–0.79, 0.12)			–0.29 (–0.73, 0.15)	
8–10		20,443		–0.53 (–0.94, –0.11)			–0.49 (–0.90, –0.08)	
≥ 11		9,526		–0.91 (–1.47, –0.36)			–0.92 (–1.47, –0.38)	
Smoking					0.15			0.08
Never		15,824		–0.85 (–1.32, –0.38)			–0.85 (–1.32, –0.39)	
Former		12,258		–0.33 (–0.83, 0.18)			–0.27 (–0.76, 0.22)	
Current		16,354		–0.44 (–0.86, –0.02)			–0.39 (–0.80, –0.03)	
Outdoor temperature (°C)					0.04			0.02
≤ 15		39,163		–0.63 (–1.00, –0.28)			–0.61 (–0.96, –0.26)	
> 15		5,273		0.02 (–0.61, 0.65)			0.13 (–0.50, 0.76)	
Area					0.37			0.47
Copenhagen city		11,486		–0.65 (–1.08, –0.23)			–0.56 (–0.99, –0.13)	
Aarhus city		13,028		–0.62 (–1.20, –0.04)			–0.68 (–1.26, –0.11)	
Copenhagen/Aarhus surroundings		19,922		–0.24 (–0.78, 0.30)			–0.27 (–0.78, 0.24)	
Municipality SES*a*					0.98			0.99
Low		6,268		–0.54 (–1.23, 0.15)			–0.52 (–1.17, 0.14)	
Medium		28,573		–0.55 (–0.93, 0.16)			–0.50 (–0.88, 0.12)	
High		9,595		–0.49 (–1.08, 0.10)			–0.48 (–1.07, 0.11)	
Prior diagnosis of cardiovascular disease*b*					0.09			0.03
Yes		1,006		0.74 (–0.77, 2.24)			1.08 (–0.40, 2.56)	
No		43,430		–0.57 (–0.91, –0.22)			–0.54 (–0.88, –0.20)	
Analyses adjusted by age, sex, center, calendar year, area, smoking status, BMI, length of school attendance, municipality SES, alcohol intake, intake of fruit and vegetables, physical activity, traffic noise, season, temperature, and relative humidity. **a**Based on municipality information on education, work market affiliation, and income. **b**A diagnosis of myocardial infarction and/or stroke before enrollment.								

Of the 50,721 participants included in the analyses of prevalent self-reported hypertension at baseline, 8,201 reported that they had been diagnosed with hypertension. Long-term exposure to NO_x_ was inversely associated with the prevalence of hypertension [evaluated as odds ratios (ORs); Table 4], with similar associations estimated for corresponding exposures to NO_2_ and NO [see Supplemental Material, Table 2 (http://dx.doi.org/10.1289/ehp.1103631)]. The presence of a major road within 50 or 100 m of the residence seemed to be associated with a lower prevalence of hypertension, whereas there was no evidence of an association with traffic load.

**Table 4:** Associations between NO_x_ and traffic at the residence and risk for prevalent and incident hypertension.

Hypertension at baseline*a*			Hypertension at follow-up		
Exposure	No. cases	OR (95% CI)*b*	No. cases	IRR (95% CI)*c*
NO_x_ 5-year mean (µg/m^3^)*d*^,e^								
< 16.1		1,317		1.00		799		1.00
16.1–19.0		2,130		0.95 (0.88, 1.04)		799		1.11 (1.00, 1.24)
19.0–26.6		2,377		0.98 (0.89, 1.07)		799		1.03 (0.91, 1.16)
> 26.6		2,377		0.89 (0.80, 0.99)		798		1.06 (0.92, 1.23)
Linear trend per doubling		8,201		0.96 (0.91, 1.00)		3,195		1.01 (0.94, 1.09)
NO_x_ 1-year mean (µg/m^3^)*d*^,e^								
< 14.3		330		1.00		798		1.00
14.3–18.2		2,423		1.00 (0.87, 1.17)		800		1.03 (0.92, 1.15)
18.2–25.2		2,763		1.01 (0.87, 1.15)		798		1.04 (0.92, 1.17)
> 25.2		2,685		0.97 (0.83, 1.14)		799		1.03 (0.89, 1.19)
Linear trend per doubling		8,201		0.95 (0.91, 1.00)		3,195		1.01 (0.95, 1.08)
Major road*f* within 50 m*g*								
No		668		1.00		2,958		1.00
Yes		7,532		0.95 (0.85, 1.05)		237		1.13 (0.97, 1.32)
Major road*f* within 100 m*g*								
No		1,386		1.00		2,664		1.00
Yes		6,814		0.93 (0.86, 1.01)		531		1.00 (0.89, 1.12)
Traffic load within 200 m (10^3^ vehicle km/day)*e*^,g^								
< 0.87		1,961		1.00		798		1.00
0.87–2.53		2,041		1.03 (0.96, 1.10)		799		1.01 (0.92, 1.12)
2.53–6.48		2,048		1.03 (0.95, 1.10)		800		1.07 (0.96, 1.18)
> 6.48		2,151		0.99 (0.91, 1.09)		798		1.02 (0.90, 1.16)
Linear trend per doubling		8,201		1.01 (0.99, 1.02)		3,195		1.01 (0.98, 1.03)
IRR, incidence rate ratio. **a**Cohort participants who in the baseline questionnaire answered that they suffered from hypertension. **b**Adjusted for age, sex, center, calendar year, area, smoking status, BMI, length of school attendance, municipality SES, alcohol intake, intake of fruit and vegetables, physical activity, traffic noise, season, temperature, and relative humidity. **c**Stratified by sex and calendar year and adjusted for smoking status, BMI, length of school attendance, municipality SES, area, alcohol intake, intake of fruit and vegetables, physical activity, and traffic noise. **d**Time-weighted average concentration of NO_x_ at residences for 1 and 5 years preceding hypertension/censoring. **e**The cutoff points between exposure groups were the 25th, 50th, and 75th percentiles for the follow-up cases at time of diagnosis. **f**More than 10,000 vehicles per day. **g**At enrollment.								

*Follow-up for hypertension.* Of the 45,271 persons that filled out the follow-up questionnaire, we excluded 7,110 with hypertension at or prior to enrollment, 1,841 participants with missing or contradictory answers to the hypertension questions, 2,897 with incomplete residential address information, and 148 with missing information on covariates, leaving a study base of 33,275 participants with an average follow-up period of 5.3 years. Among these, 3,195 participants reported that they had been diagnosed with hypertension within the follow-up period.

Table 1 shows the distribution of baseline characteristics in the study population. The distribution of baseline characteristics among the 33,275 participants followed up for hypertension was very similar to the distributions in the baseline study cohort.

We found no clear associations between exposure to traffic-related air pollution and risk for self-reported hypertension in the subset of participants who responded at the follow-up survey (evaluated as IRRs; Table 4). In analyses of NO_x_, point estimates were slightly elevated, but CIs included the null. Estimates did not demonstrate monotonic dose–response relations with increasing quartiles of exposure. Participants who lived within 50 m of a major road had a 13% higher risk for hypertension (95% CI: 0.97%, 1.32%). Exclusion of participants with a history of myocardial infarction (*n* = 335) or stroke (*n* = 206) at baseline resulted in only minor changes in estimated associations (data not shown). Also with regard to exposure to long-term NO_2_ and NO, no clear associations were found between exposure and risk for hypertension [see Supplemental Material, Table 2 (http://dx.doi.org/10.1289/ehp.1103631)].

## Discussion

Long-term exposure to traffic-related air pollution was inversely associated with systolic and DBP and the prevalence of self-reported hypertension in a cross-sectional design, whereas long-term exposure to traffic-related air pollution was not associated with the risk of self-reported hypertension during approximately 5 years of follow-up.

*Strengths and limitations.* Strengths included the large study population, with detailed information on potential confounders. Furthermore, access to residential address histories improved estimation of long-term air pollution. In addition, we adjusted for exposure to road traffic noise, which potentially is associated with traffic-related air pollution (de Kluizenaar et al. 2007; Sørensen et al. 2011) and has been associated with BP and hypertension (Babisch 2006). However, we cannot rule out residual confounding, for example, by individual SES or intake of sodium or potassium.

Although the dispersion models used to estimate long-term exposures to air pollution in the present study have been successfully validated and applied (Andersen et al. 2011; Berkowicz et al. 2008; Ketzel et al. in press; Raaschou-Nielsen et al. 2000), such estimates are inevitably associated with some degree of uncertainty, which would result in exposure misclassification. However, such misclassification should be nondifferential with respect to BP and hypertension.

A limitation of the study of measured BP at baseline part of this study is the cross-sectional design. Although we have adjusted for many possible confounders, associations should be confirmed using a longitudinal design with repeated measures. Results of previous studies of air pollution and BP measured at different points in time have been inconsistent, with some studies reporting positive associations (de Paula et al. 2005; Dvonch et al. 2009; Zanobetti et al. 2004), and others reporting inverse associations (Brauer et al. 2001; Ebelt et al. 2005; Ibald-Mulli et al. 2004) without clear relations between the results observed and the design of the study.

The measurement of systolic and DBP in our study was standardized but did not follow standard clinical protocols for diagnosing hypertension, which require several measurements of BP. We repeated measurements only if SBP was ≥ 160 mmHg or DBP was ≥ 95 mmHg, and used the lower of the two measurements, which may have resulted in a systematic bias toward lower values in participants with higher BPand could have biased the BP estimate toward an inverse association. When we restricted the sample to participants with normal BP values, who were less likely to have had repeated BP measurements, inverse associations were less pronounced but still evident between long-term NO_x_ and BP. However, it is not possible to determine whether or how much differences observed after restriction reflect a reduction in misclassification of the BP measurements versus selection bias caused by limiting the analysis to potentially less susceptible participants.

Our prospective study of hypertension also has some limitations. First, information on hypertension was self-reported, and the actual number of hypertensive participants is probably underestimated. Therefore, a number of participants who were actually hypertensive at baseline were falsely included as nonhypertensive. Such misclassification may have led to a systematic bias; for example, if underreporting was most prominent in low-SES groups, who are often exposed to the highest levels of air pollution, risk estimates may have been biased downward. Also, all participants had their BP taken at baseline, and it is very likely that those with a high BP measurement would subsequently have been examined further by their physician. Therefore, many with undiagnosed hypertension at enrollment will potentially be diagnosed immediately after enrollment. However, exclusion of the cases diagnosed within the first year (27%) from the analyses did not change the estimates markedly, indicating that this did not result in a systematic bias.

*Systolic and DBP.* Our finding of a weak inverse association between air pollution and BP was robust across different model specifications. Furthermore, both long-term and short-term air pollution exposures, as well as two proxy measures of traffic exposure, were inversely associated with BP.

Two studies of longer-term exposure to air pollution and BP have reported positive associations (Auchincloss et al. 2008; Chuang et al. 2011). Results of previous studies of short-term exposures and BP have been inconsistent (Brauer et al. 2001; Brook et al. 2009; Chuang et al. 2010; de Paula et al. 2005; Dvonch et al. 2009; Harrabi et al. 2006; Ibald-Mulli et al. 2004; Urch et al. 2005; Zanobetti et al. 2004). Ibald-Mulli et al. (2004) suggested that a possible mechanism for a decrease in BP caused by exposure to air pollution could be a shift in sympathovagal balance due to an increase in vagal tone. Another explanation could relate to the effect of NO as a potent vasodilator that diffuses freely across membranes. NO is present in exhaust from vehicles and is converted to NO_2_ through reaction with ozone. Because ozone is generated from oxygen reacting with sunlight, NO is usually present in lowest concentrations during summer. NO, NO_2_, and their sum, NO_x_, are highly correlated (Hertel et al. 2001; Ketzel et al. 2003), and it is therefore extremely difficult to disentangle effects of the three exposures. A closer look at the results reported by Auchincloss et al. (2008) indicates that the positive association between long-term air pollution exposure and BP was evident only in the warmer season (> 10°C); whereas at temperatures < 10°C, the association tended to be negative although not statistically significantly. Similarly, we found that the inverse association between air pollution and BP was present only at temperatures < 15°C. The concentration of NO_x_ is rather constant during a year, but during summer the contribution of NO is reduced because of higher ozone concentrations. This and other factors that could influence NO concentrations, such as geography and season, might also explain differences in results among studies of different populations.

Our results suggest that among patients with a previous diagnosis of cardiovascular disease, long-term exposure to NO_x_ might be positively associated with BP, indicating that these patients might be a susceptible group. This analysis was, however, based on relatively few patients with cardiovascular disease.

*Hypertension.* We found inconsistent associations of long-term air pollution with hypertension. In the cross-sectional analysis of self-reported hypertension at baseline, we saw a small inverse association, consistent with the results of the BP analysis. However, exposure was not inversely associated with incident self-reported hypertension, and results could indicate a slight positive association, although estimates did not indicate a monotonic dose-dependent relationship. Studies using validated hypertension as outcome are necessary to disentangle possible sources of bias.

To our knowledge, this is the first study to estimate effects of long-term air pollution on the incidence of hypertension. A few previous studies have reported that short-term air pollution was associated with emergency department visits for hypertension (Guo et al. 2010a, 2010b) and that long-term air pollution was positively associated with prevalence of self-reported hypertension (Johnson and Parker 2009). Exposure to air pollution has been associated with increased inflammation and oxidative stress, as well as endothelial dysfunction (Brook et al. 2010; Hoffmann et al. 2009), which may contribute to the development and progression of atherosclerosis and risk of hypertension. Because most studies have focused on PM_2.5_ and not NO and NO_2_, direct comparisons are difficult. In contrast to well-known vasodilatory effects of NO, PM mixtures are extremely variable and may have very different physiological effects depending on the predominant constituents.

## Conclusions

Long-term exposure to traffic-related air pollution was associated with a slightly lower BP but was not consistently associated with self-reported hypertension.

## Supplemental Material

(135 KB) PDF
