# Supplementary material for: Long-Term Exposure to Traffic-Related Air Pollution Associated with Blood Pressure and Self-Reported Hypertension in a Danish Cohort
Source: Environ Health Perspect. 2012 Jan 3;120(3):418–24. doi: 10.1289/ehp.1103631 (PMC3295339; doi:10.1289/ehp.1103631)
Supplement: (135 KB) PDF [file ehp.1103631.s001.pdf]

Supplement Material

Long-term Exposure to Traffic-Related Air Pollution, Blood Pressure and Self-Reported Hypertension in a Danish Cohort

Mette Sørensen, Barbara Hoffmann, Martin Hvidberg, Matthias Ketzel, Steen Solvang Jensen, Zorana Jovanovic Andersen, Anne Tjønneland, Kim Overvad, Ole Raaschou-Nielsen

Table of Contents

SoundPLAN	page 2
References	page 2
Supplement Table 1	page 3
Supplement Table 2	page 5

SoundPLAN

This noise calculation program implements the joint Nordic prediction method for road traffic noise, which has been the standard method for noise calculation in Scandinavia during many years (Bendtsen 1999; Danish EPA and Danish Road Directorate 1998). The input variables were: 1) geographical coordinates and height of each dwelling over terrain, 2) road lines with information on annual average daily traffic, traffic composition, traffic speed and road type (motorway, rural highway, road wider than 6 m, and other road), 3) building polygons. We assumed that the terrain was flat, which is a reasonable assumption in Denmark, and that urban areas, roads, and areas with water were hard surfaces whereas all other areas were acoustically porous. No information was available on noise barriers. Road traffic noise was calculated as L_{Aeq} at the most exposed façade of the dwelling at each enrolment address for the day (07:00-19:00 h), evening (19:00-22:00 h) and night (22:00-07:00 h) and expressed as L_{den}.

References

Bendtsen H. 1999. The Nordic prediction method for road traffic noise. Sci Total Environ 235:331-338.

Danish EPA Danish Road Directorate. 1998. Beregningsmodel for vejtrafikstøj, revideret 1996. rapport nr 178.

Supplement table 1 Associations between concentrations of NO_2 and NO and traffic at the residence and the systolic and diastolic blood pressure at enrolment

**Exposure**	N	**Differences in systolic blood pressure (mm Hg) (95% confidence interval)** [a]	**Differences in diastolic blood pressure (mm Hg) (95% confidence interval)** [a]
NO_2 5-year mean ($\mu g/m^3$) [b,c]			
< 14.0	11,109	0.00	0.00
14.0 – 15.6	11,109	-0.30 (-0.82; 0.23)	0.00 (-0.27; 0.28)
15.6 – 20.5	11,109	-0.34 (-0.93; 0.26)	-0.15 (-0.47; 0.16)
> 20.5	11,109	-0.86 (-1.59; -0.13)	-0.36 (-0.75; 0.02)
Linear trend per doubling	44,436	-1.00 (-1.64; -0.37)	-0.53 (-0.86; -0.19)
NO_2 1-year mean ($\mu g/m^3$) [b,c]			
< 14.2	11,116	0.00	0.00
14.2 – 16.3	11,102	-0.36 (-0.91; 0.18)	0.01 (-0.28; 0.29)
16.3 – 20.4	11,109	-0.43 (-1.04; 0.18)	-0.20 (-0.52; 0.13)
> 20.4	11,109	-0.95 (-1.68; -0.23)	-0.43 (-0.81; -0.04)
Linear trend per doubling	44,436	-1.21 (-1.87; -0.54)	-0.55 (-0.90; -0.20)
NO_2, 3-days mean[b,d]			
< 10.7	5,360	0.00	0.00
10.7-13.5	5,419	-0.93 (-1.64; -0.21)	-0.41 (-0.79; -0.03)
13.5-17.5	5,353	-1.26 (-2.00; -0.53)	-0.76 (-1.15; -0.37)
> 17.5	5,375	-0.78 (-1.53; -0.04)	-0.16 (-0.56; 0.23)
Linear trend per doubling	21,507	-0.69 (-1.22; -0.17)	-0.28 (-0.56; -0.00)
NO 5-year mean ($\mu g/m^3$) [b,c]			
< 2.9	11,109	0.00	0.00
2.9 – 4.0	11,109	-0.24 (-0.76; 0.27)	-0.12 (-0.36; 0.16)
4.0 – 9.1	11,109	-0.57 (-1.14; 0.01)	-0.52 (-0.82; -0.21)
> 9.1	11,109	-0.87 (-1.57; -0.17)	-0.55 (-0.92; -0.18)
Linear trend per doubling	44,436	-0.28 (-0.46; -0.09)	-0.14 (-0.23; -0.04)
NO 1-year mean ($\mu g/m^3$) [b,c]			
< 2.8	11,116	0.00	0.00
2.8 – 3.6	11,102	-0.36 (-0.89; 0.16)	-0.07 (-0.34; 0.21)
3.6 – 7.9	11,109	-0.55 (-1.15; 0.04)	-0.39 (-0.70; -0.07)
> 7.9	11,109	-0.67 (-1.42; 0.03)	-0.38 (-0.77; 0.00)
Linear trend per doubling	44,436	-0.25 (-0.44; -0.07)	-0.12 (-0.22; -0.02)
NO, 3-days mean[b,d]			
< 3.8	5,360	0.00	0.00
3.8-6.0	5,419	-0.65 (-1.35; 0.06)	-0.28 (-0.66; 0.09)
6.0-10.3	5,353	-0.42 (-1.16; 0.31)	-0.15 (-0.54; 0.24)
> 10.3	5,375	-0.83 (-1.63; -0.04)	-0.39 (-0.81; 0.04)
Linear trend per doubling	21,507	-0.25 (-0.54; 0.04)	-0.13 (-0.28; 0.03)

Supplement Table 1 continued

[a] Adjusted for age, sex, centre, calendar-year, area, smoking status, BMI, length of school attendance, municipality SES, alcohol intake, intake of fruit and vegetables, physical activity, traffic noise, season, temperature and humidity

[b] The cut-off points between exposure groups were the 25th, 50th and 75th percentiles for all participants at the time of inclusion into the cohort

[c] Time-weighted average concentration of NO_2 /NO at residences 1 and 5 years preceding enrolment

[d] Based only on participants from the Copenhagen centre. The analysis of the 3-days mean of NO_2 /NO was also adjusted by the preceding 1-year mean concentration of NO_2 /NO

Supplement Table 2 Associations between NO_2 and NO and traffic at the residence and risk for prevalent and incident hypertension

Exposure	Hypertension at baseline[a] N cases	Hypertension at baseline[a] Odds Ratio (95% confidence interval)[b]	Hypertension at follow-up N cases	Hypertension at follow-up Incidence rate ratio (95% confidence interval)[c]
NO_2 5-year mean (µg/m^3) [d,e]				
< 13.1	1359	1.00	798	1.00
13.1 – 15.1	2016	0.98 (0.91-1.07)	799	1.07 (0.96-1.19)
15.1 – 19.6	2161	0.96 (0.87-1.05)	799	1.08 (0.96-1.22)
> 19.6	2665	0.97 (0.87-1.08)	799	0.99 (0.91-1.14)
Linear trend per doubling	8201	0.93 (0.85-1.01)	3195	1.00 (0.88-1.14)
NO_2 1-year mean (µg/m^3) [d,e]				
< 11.5	103	0.84 (0.67-1.05)	799	1.00
11.5 – 14.5	2061	1.00	798	0.93 (0.83-1.05)
14.5 – 18.8	2787	0.94 (0.87-1.01)	800	0.97 (0.86-1.09)
> 18.8	3250	0.98 (0.90-1.07)	798	0.90 (0.78-1.05)
Linear trend per doubling	8201	0.91 (0.83-1.00)	3195	0.97 (0.87-1.08)
NO 5-year mean (µg/m^3) [d,e]				
< 2.7	832	1.00	799	1.00
2.7 – 3.6	2506	0.97 (0.89-1.07)	798	1.06 (0.95-1.18)
3.6 – 7.9	2631	0.97 (0.87-1.07)	800	0.99 (0.88-1.12)
> 7.9	2232	0.92 (0.81-1.03)	798	1.03 (0.90-1.19)
Linear trend per doubling	8201	0.98 (0.95-1.00)	3195	1.01 (0.97-1.05)
NO 1-year mean (µg/m^3) [d,e]				
< 2.6	1253	1.00	798	1.00
2.6 – 3.4	2131	0.91 (0.84-0.99)	800	1.12 (1.01-1.24)
3.4 – 6.8	2561	0.92 (0.84-1.01)	799	1.06 (0.94-1.19)
> 6.8	2256	0.90 (0.81-1.01)	798	1.11 (0.97-1.26)
Linear trend per doubling	8201	0.98 (0.95-1.00)	3195	1.02 (0.98-1.06)

[a] Defined as cohort participants who in the baseline questionnaire answered that they suffered from hypertension
[b] Adjusted for age, sex, centre, calendar-year, area, smoking status, BMI, length of school attendance, municipality SES, alcohol intake, intake of fruit and vegetables, physical activity, traffic noise, season, temperature and humidity
[c] Stratified by gender and calendar year and adjusted for smoking status, BMI, length of school attendance, municipality SES, area, alcohol intake, intake of fruit and vegetables, physical activity and traffic noise
[d] Time-weighted average concentration of NO_x at residences for 1 and 5 years preceding hypertension/censoring
[e] The cut-off points between exposure groups were the 25th, 50th and 75th percentiles for the follow-up cases at the time of diagnosis
